# miR-146a regulates the crosstalk between intestinal epithelial cells, microbial components and inflammatory stimuli

**DOI:** 10.1038/s41598-018-35338-y

**Published:** 2018-11-26

**Authors:** Andrea Anzola, Raquel González, Reyes Gámez-Belmonte, Borja Ocón, Carlos J. Aranda, Patricia Martínez-Moya, Rocío López-Posadas, Cristina Hernández-Chirlaque, Fermín Sánchez de Medina, Olga Martínez-Augustin

**Affiliations:** 1 Department of Pharmacology, CIBERehd, School of Pharmacy, Instituto de Investigación Biosanitaria ibs.GRANADA, University of Granada, Granada, Spain; 2 Department of Biochemistry and Molecular Biology II, CIBERehd, School of Pharmacy, Instituto de Investigación Biosanitaria ibs.GRANADA, University of Granada, Granada, Spain

## Abstract

Regulation of miR-146a abundance and its role in intestinal inflammation and particularly in intestinal epithelial cells (IECs) has been poorly studied. Here we study the relationship between bacterial antigens and inflammatory stimuli, and miR-146a expression using IEC lines and models of colitis (trinitrobenzenesulfonic acid (TNBS), dextran sulfate sodium (DSS) and the CD4 + CD62L + T cell transfer model). Specific bacterial antigens and cytokines (LPS, flagelin and IL-1β/TNF) stimulate miR-146a expression, while peptidoglycan, muramyldipeptide and CpG DNA have no effect. Overexpression of miR-146a by LPS depends on the activation of the TLR4/MyD88/NF-kB and Akt pathways. Accordingly, the induction of miR-146a is lower in TLR4, but not in TLR2 knock out mice in both basal and colitic conditions. miR-146a overexpression in IECs induces immune tolerance, inhibiting cytokine production (MCP-1 and GROα/IL-8) in response to LPS (IEC18) or IL-1β (Caco-2). Intestinal inflammation induced by chemical damage to the epithelium (DSS and TNBS models) induces miR-146a, but no effect is observed in the lymphocyte transfer model. Finally, we found that miR-146a expression is upregulated in purified IECs from villi vs. crypts. Our results indicate that miR-146a is a key molecule in the interaction among IECs, inflammatory stimuli and the microbiota.

## Introduction

Intestinal homeostasis depends on the interaction between bacteria and the intestinal epithelium. This fact is clearly manifested in intestinal inflammatory diseases, in which dysbiosis is known to play a pathogenic role^1^. In addition, dysbiosis has been related to systemic diseases such as fatty liver disease, obesity or diabetes^2,3^. The current view is that the intestinal immune system and the microbiota maintain a collaborative alliance in which the microbiota modulates the immune system and, in turn, the latter tolerates microbiota and fights off invasive pathogenic bacteria^4^. This partnership is possible to a great extent due to non-specific receptor in innate immune cells that recognize bacterial components. Among these receptors, TLRs are the best characterized, together with the retinoic acid-inducible gene I (RIG-I)-like receptors and NLRs^5,6^.

Intestinal epithelial cells (IECs) are considered components of the intestinal innate immune system that play a major role in the cross-talk with the microbiota and the development of tolerance. In fact, IECs express TLRs, but under homeostatic conditions expression is low, and the effects of their ligands are correspondingly attenuated^7^. Nevertheless, under inflammatory conditions TLR expression is increased and contributes to inflammation and immune tolerance^8^.

In the last few years microRNAs (miRNAs) have been shown to be involved in the regulation of the inflammatory response by microbiota-derived antigens. These small non-coding RNAs regulate gene expression and, as such, are involved in the pathogenesis of several diseases^9,10^. A general mechanism of action of miRNAs involves the regulation of protein expression both at the transcriptional and translational level. Among the miRNAs associated to the inflammatory response and the microbiota, miR-146a is strongly induced by endotoxin through the stimulation of TLR4 in different cell types^11,12^. Furthermore, it has been shown to participate in the regulation of the immune response limiting it to prevent overstimulation^13–15^. The response of miR-146a knock out mice to endotoxin has given proof of the negative feedback regulatory loop that controls pro-inflammatory signaling, as miR-146a knock out mice are hyper-responsive to lipopolysaccharide. Thus, miR-146a is considered a negative effector of the innate immune response^14^. Although there are not many studies assessing the role of miR-146a in intestinal inflammation, a study indicated that miR-146a also protects against intestinal ischemia/reperfusion injury^16^. Nevertheless, later findings have introduced controversy regarding the role of miRNA-146a in the intestinal inflammation. Thus a recent study showed that knock out animals are resistant to dextran sulfate sodium (DSS) induced colitis, a chemical model in which the intestinal barrier function is disrupted. The current explanation for this apparent contradiction is based on the ability of miR-146a to inhibit both inflammatory and intestinal barrier related genes^14^. In this study we aimed to better understand the regulation of miR-146a in intestinal inflammation studying its expression in three animal models of colitis, which differ in the involvement of the intestinal mucosal barrier and, accordingly, in the level of contact with the intestinal microbiota. In addition, we have used the DSS model in TLR2 and TLR4 knock out mice to study the involvement of bacterial antigens in the expression of miR-146a.

Because miR-146a is expressed in hematopoietic cells, and these cells are key mediators of the immune response, most studies have been restricted to these cell types (or to whole tissue) and the role of miR-146a in the immune response mediated by IECs has been poorly studied. Our second objective in this study aims therefore to further report the role of miR-146a in intestinal inflammation and the maintenance of immune tolerance by IECs. We studied the effect of bacterial antigens and cytokines on the expression of miR-146a using IEC lines, and overexpressed miR-146a in IECs to explore the hypothesis that it may contribute to prevent overstimulation of the immune response. Characterization of the function of miR-146a in intestinal inflammation and tolerance will contribute to better understand these processes and their contribution to the pathogenesis of inflammatory diseases like inflammatory bowel disease and even of systemic diseases in which a role for the intestinal microbiota has been suggested.

## Results

### Proinflammatory cytokines and the bacterial components LPS and flagellin induce miR-146a expression in intestinal epithelial cells

**Figure 1:**
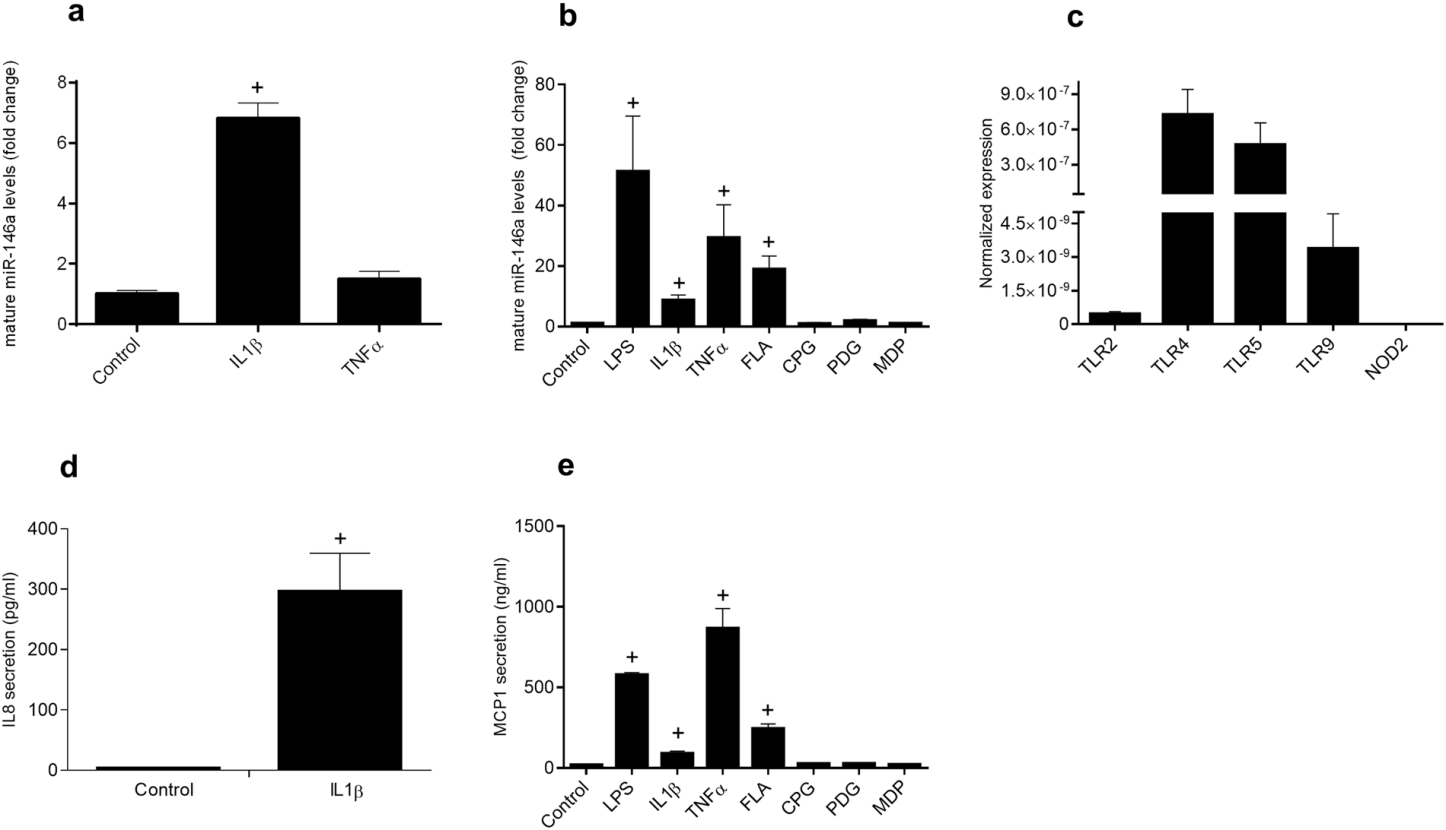
miR-146a expression in Caco-2 and IEC18 cells in response to proinflammatory cytokines and bacterial antigens. Confluent cell monolayers were incubated with LPS (1 μg/ml), flagellin (FLA, 100 ng/ml), CpG DNA (50 nM), peptidoglycan (PDG, (2 μg/mL), muramyl dipeptide (MDP, 5 μg/ml), IL-1β (10 ng/ml) or TNFα (10 ng/ml) for 24 h. (**a**) mature miR-146a in caco-2 cells and (**b**) mature miR-146a in IEC18 cells. U6 was used as reference gene for miR-146a determinations. (**c**) Relative TLR 2, 4, 5 and 9 and NOD-2 expression in IEC18 cells assessed by RT-qPCR. rRNA 18 S subunit was used as a reference gene (Ct: 6.0 ± 0.5). (**d**) IL-8 secretion in the cells culture medium of Caco-2 cells and (**e**) MCP-1 secretion in the cell culture medium of IEC18 cells measured by ELISA. *p < 0,05 vs control group (n = 3–8).

Caco-2 cells (human adenocarcinoma colonic cells) mature miR-146a expression was sharply upregulated in response to IL-1β but not to TNF (Fig. 1a, p = 0.1 for the latter). Quiescent IEC18 cells (nontumoral rat ileal cells) displayed low expression of mature miR-146a, which shifted to substantially increased levels after stimulation with LPS (a TLR4 ligand), flagellin (a TLR5 ligand), TNF or IL-1β (Fig. 1b). Conversely, no significant effect of CpG DNA (a TLR9 ligand), peptidoglycan (a TLR2 ligand), or muramyldipeptide (a NOD2 ligand) was detected. The expression of TLR 2, 4, 5 and 9 and NOD2 in IEC18 cells was also studied and was directly correlated with the observed stimulation of miR-146a, with TLR4 and TLR5 showing the highest expression levels (Fig. 1c). As a positive control of the effect of IEC stimulation IL-8 was measured in Caco-2 cells (Fig. 1d) and MCP-1 in IEC18 (Fig. 1e), as representative cytokines induced by immune activation in epithelial cells. The pattern of cytokine secretion paralleled that of miR-146a induction, i.e. Caco-2 cells showed increased IL-1β-evoked IL-8 production (Fig. 1d) and IEC18 cells exhibited a positive response to LPS, flagellin, TNF and IL1β, but not to other stimuli (Fig. 1e).

### Signaling through TLR4/MyD88/NF-kB-Akt induces miR-146a expression in intestinal epithelial cells

**Figure 2:**
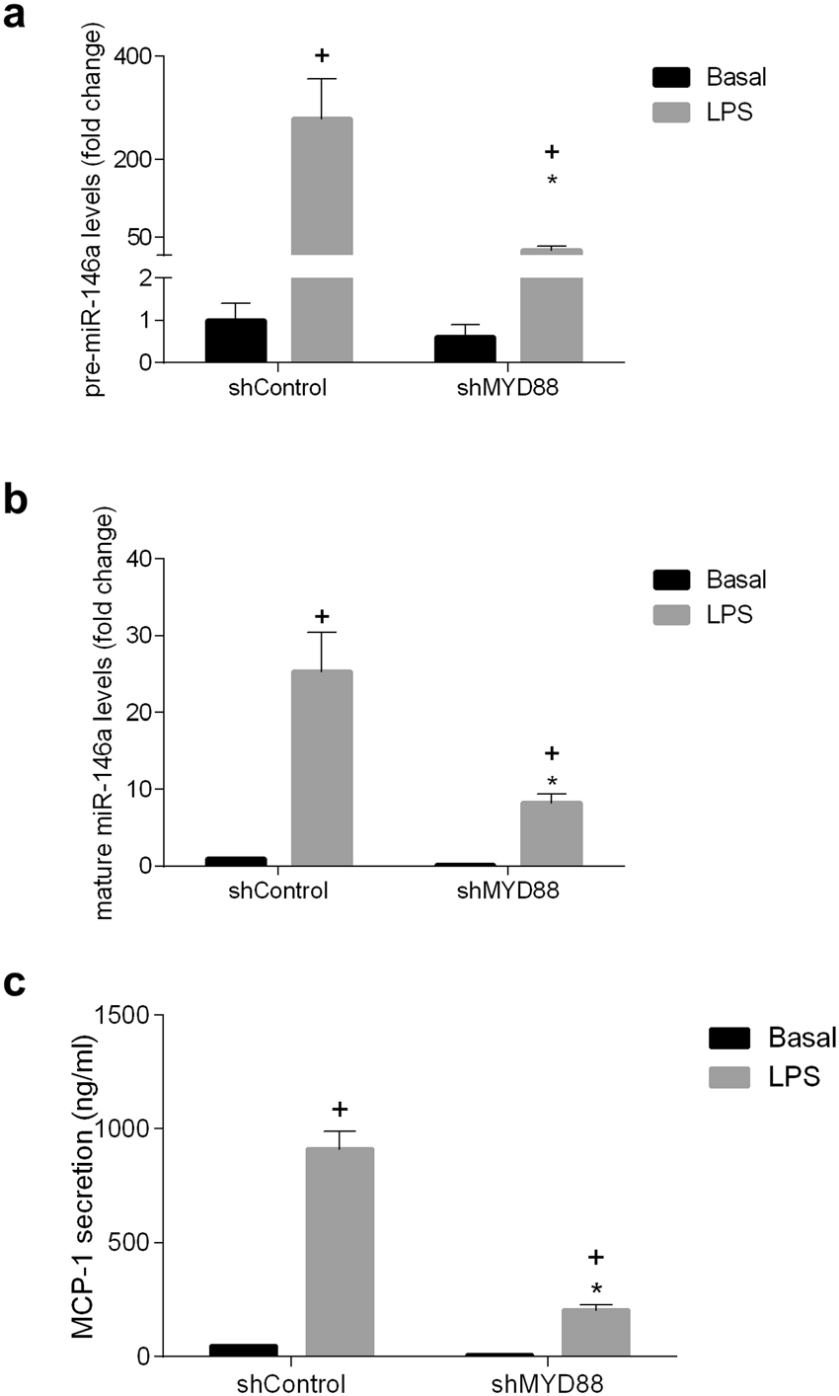
Effect of MYD88 gene silencing in IEC18 cells with siRNAs on the expression of pre-mir-146a and mature miR-146a and the secretion of MCP-1. (**a**) Expression of pre-mir-146a and (**b**) mature miR-146a assessed by RT-qPCR and (**c**) secretion of MCP-1 measured by ELISA, in basal conditions and after stimulation with LPS (1 μg/ml). U6 were used as reference gene for pre-mir-146a and mature miR-146a. ^+^p < 0.05 vs sh Control, *p < 0,05 vs sh Control +LPS. Data are representative of two independent experiments (n = 4).

**Figure 3:**
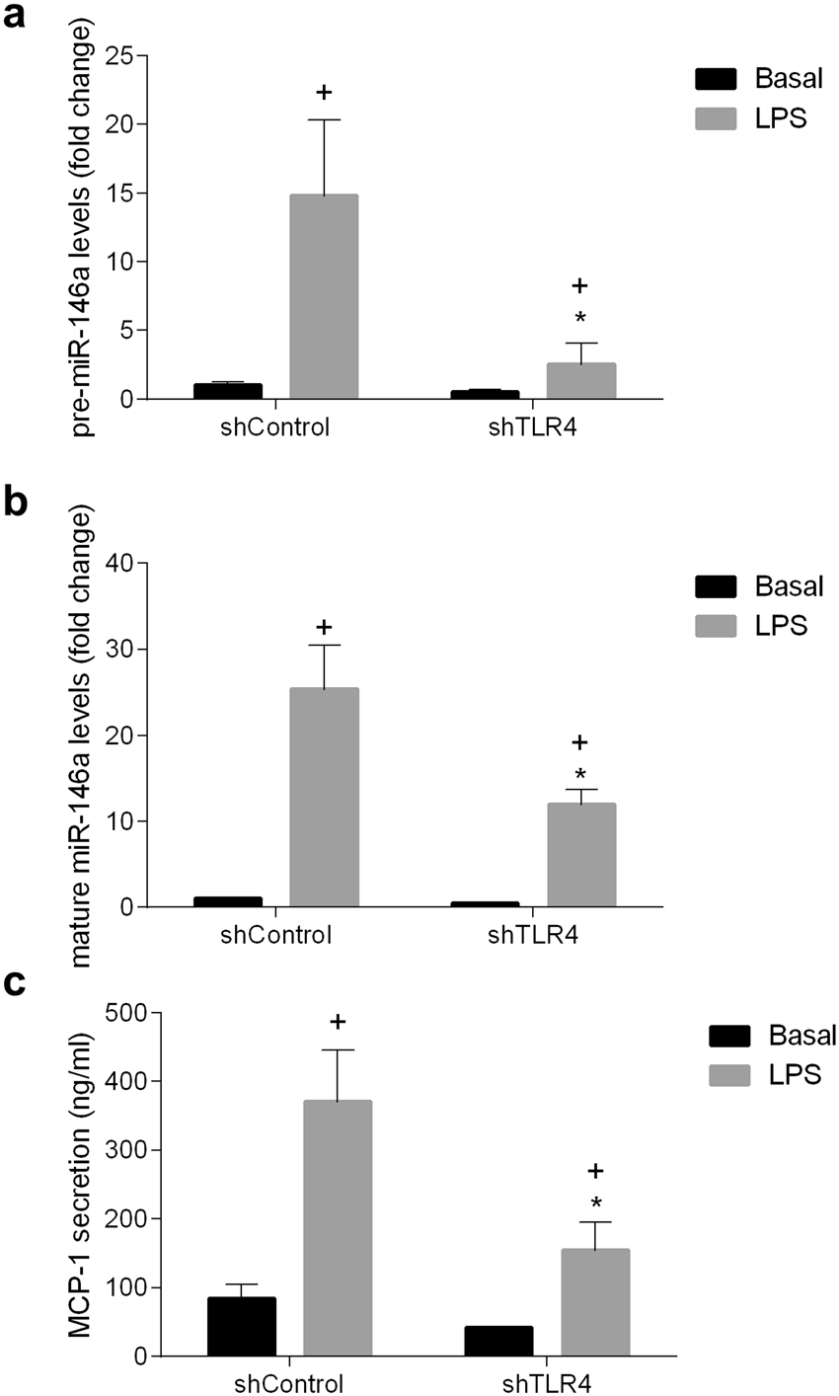
Effect of TLR4 gene silencing in IEC18 cells with siRNAs on the expression of pre-mir-146a and mature miR-146a and the secretion of MCP-1. (**a**) Expression of pre-mir-146a and (**b**) mature miR-146a assessed by RT-qPCR and (**c**) secretion of MCP-1 measured by ELISA, in basal conditions and after stimulation with LPS (1 μg/ml). U6 were used as reference gene for pre-mir-146a and mature miR-146a. ^+^p < 0.05 vs sh Control, *p < 0,05 vs sh Control +LPS. Data are representative of two independent experiments (n = 4).

MyD88 is a TLR adapter protein used by most TLRs to activate transcription factor NF-κB and mitogen activated protein kinases (MAPK), with the consequent induction of proinflammatory cytokines. AKT may be also activated after TLR4 stimulation by the product of phosphatidylinositol-3-kinase (PI3K), phosphatidylinositol-3-phosphate (PIP3). To confirm that TLR ligand evoked induction of miR-146a depends on MyD88, gene knockdown with interference RNA technology was applied to IEC18 cells. In order to investigate possible regulation of the processing of pre-mir-146a to mature miR-146a both were measured. Silencing MyD88 expression in IEC18 cells resulted in the inhibition of both pre-mir-146a and mature miR-146a expression, associated with a decrease of MCP-1 induction by LPS (Fig. 2a–c). Trends observed in pre-mir-146a and mature miR-146a indicated parallel patterns with the different challenges, indicating a transcriptional regulation. In addition, when TLR4 expression was similarly knocked down in IEC18 cells, it also resulted in attenuated LPS evoked MCP-1 release and miR-146a expression (Fig. 3a–c).

**Figure 4:**
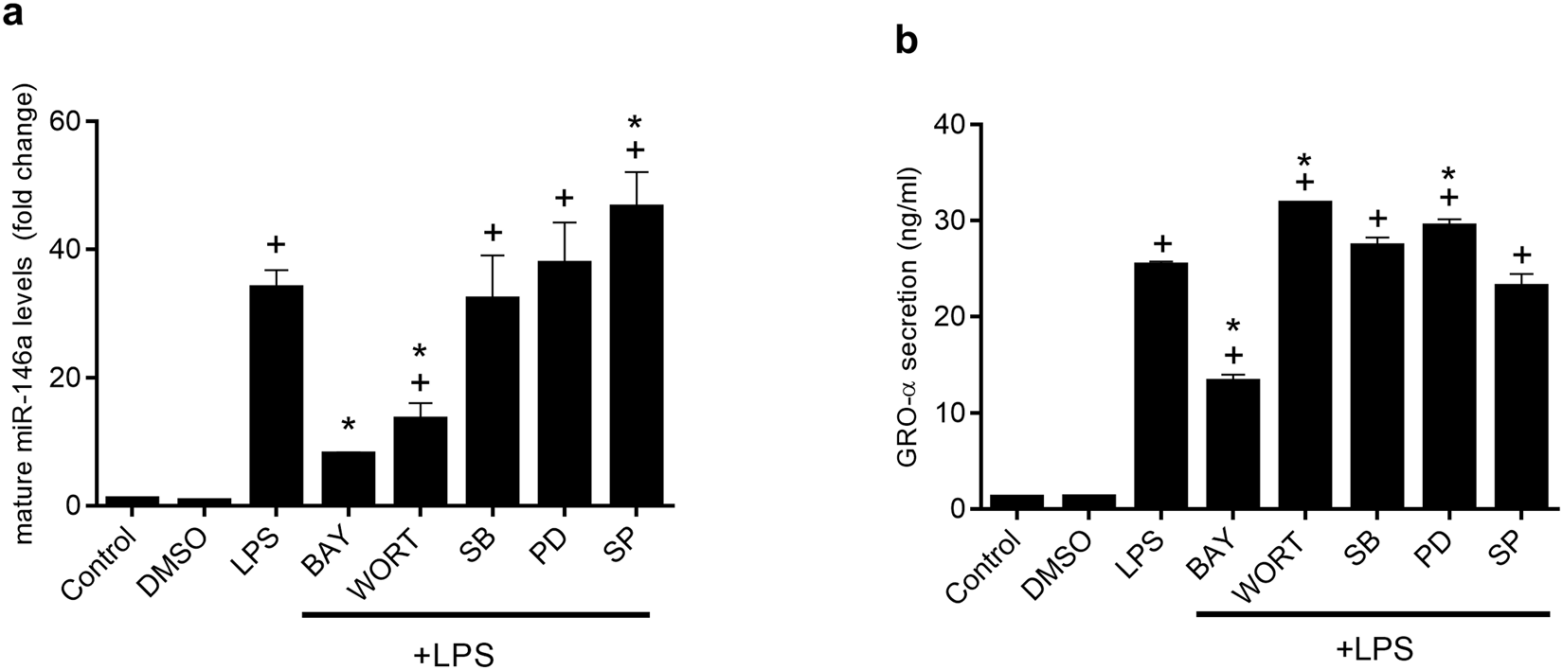
Signal transduction pathway involved in the effect of LPS in IEC18 cells. Effect of specific inhibitors, including Bay 11-7082 (10 µM) (I*κ*Bα phosphorylation), wortmannin (1 µM) (phosphatidylinositol 3 kinase, PI3K), SB203580 (10 µM) (p38 MAPK), PD98059 (10 µM) (ERK1/2 MAPK) and SP600152 (10 µM) (JNK MAPK), on the expression of mature miR-146a (**a**) and GROα (**b**). Mature miR-146a and GROα expression was studied by RT-qPCR using U6 or 18 S as reference gene, respectively. ^+^p < 0.05 vs Control, *p < 0,05 vs LPS. Data are representative of two independent experiments (n = 4).

To study the involvement of NF-kB, MAPK and AKT in the induction of miR-146a by LPS, inhibitors of these signal transduction pathways were added to cell culture before LPS stimulation, and mature miR-146a was measured by PCR (Fig. 4a). Our results show that inhibition of NF-kB downregulates miR-146a expression. The inhibition of PI3K caused a similar degree of inhibition, indicating the involvement of the AKT pathway. In contrast, inhibition of MAPKs p38 or ERK1/2 did not have any effect on LPS induced miR-146a expression, while a slight increase was observed when JNK was inhibited. As a control of the effect of IEC stimulation, GROα expression was determined. There was a close correlation of GROα levels and miR-146a expression, with the exception of cells treated with LPS+ wortmannin, which exhibited diminished miR-146a but no inhibition of cytokine production (Fig. 4b).

### Overexpression of miR-146a inhibits cytokine production in response to LPS and IL-1β in intestinal epithelial cells

**Figure 5:**
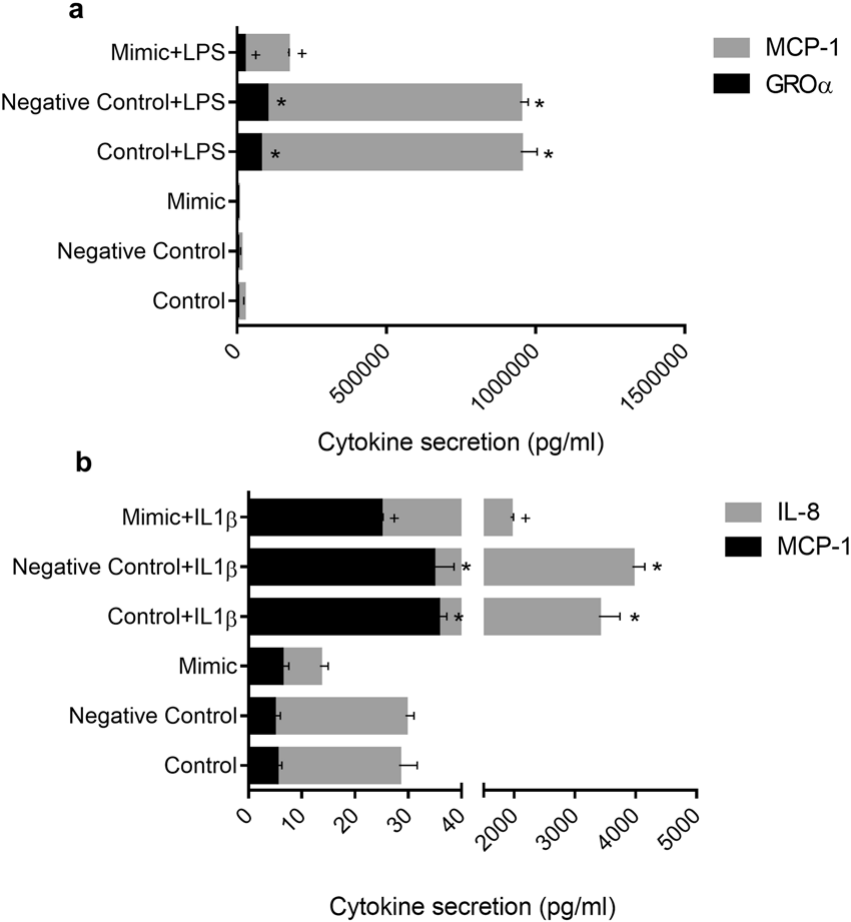
Effect of miR-146a overexpression on cytokine production in IEC18 and Caco-2 cells. The effect of mimics was studied measuring in the culture medium the cytokine production (MCP-1 and GROα in case of IEC18 cells (**a**) and MCP-1 and IL-8 in case of Caco-2 cells (**b**)) at 24 h post-transfection. The results are expressed as mean ± SEM. *p < 0.05 vs its control; ^+^p < 0,05 vs negative control +LPS or IL-1β. Data are representative of 2 independent experiments (n = 2–3).

It has been described that overexpression of miR-146a negatively regulates inflammation, limiting the immune response^13^. In order to test the validity of this hypothesis in intestinal epithelial cells, we overexpressed miR-146a in both IEC18 and Caco-2 cells and studied the effect on cytokine production (Fig. 5). Transfection of a small RNA mimic of miR-146a was employed for this purpose. Overexpression of miR-146a in IEC18 cells inhibited the production of MCP-1 and GROα in the culture medium after 24 h of incubation, both in basal conditions and after LPS stimulation (Fig. 5a). Parallel experiments carried out in Caco-2 cells confirmed these results, with a decreased production of IL-8 and MCP-1 in cells overexpressing miR-146a after IL-1β stimulation and in basal conditions (Fig. 5b).

### miR-146a is overexpressed in chemical models of colitis that affect epithelial integrity

**Figure 6:**
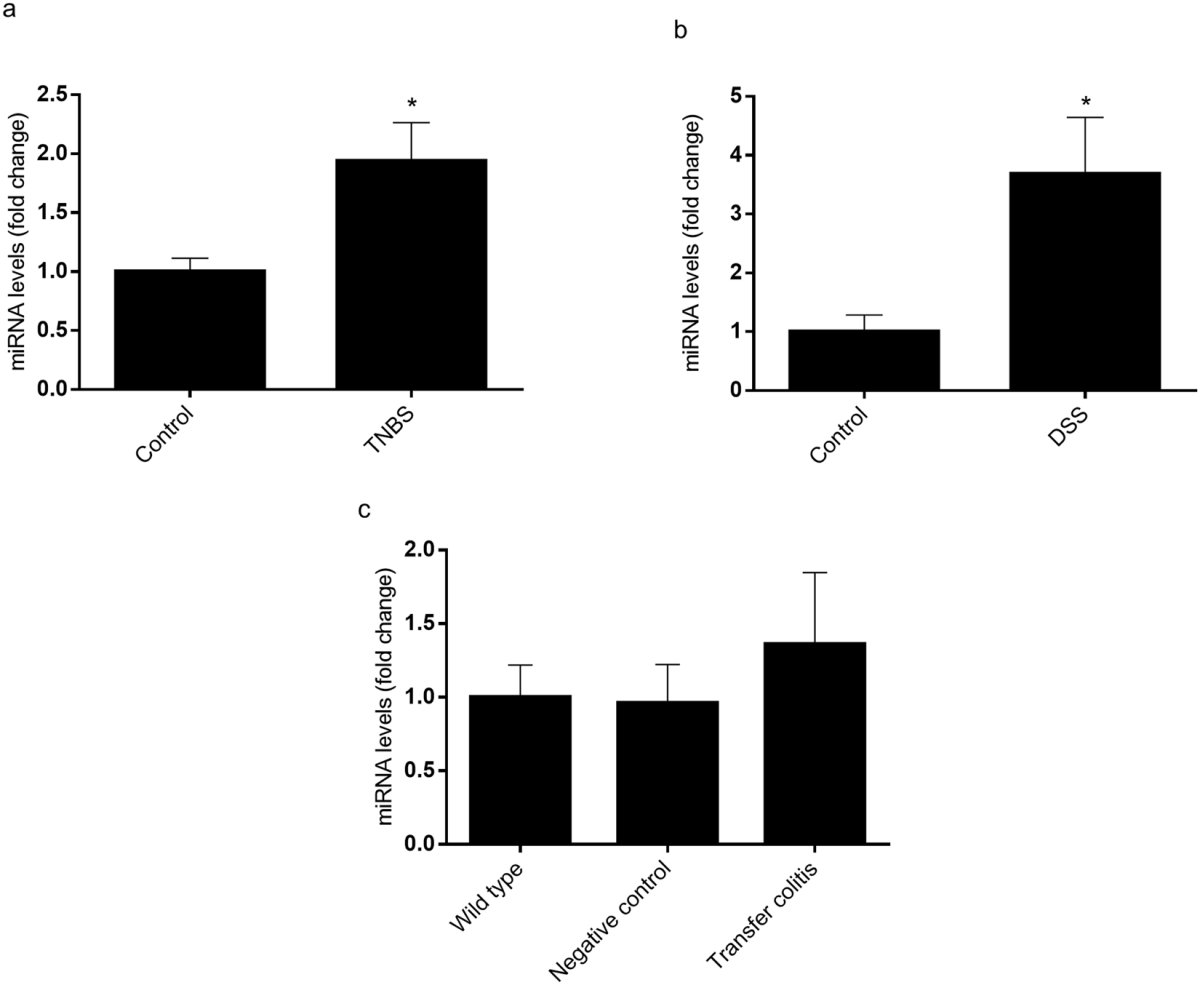
Colonic expression of mature miR-146a in animal models of colitis. Three models were used: the TNBS model of rat colitis (**a**), the DSS model of mice colitis (**b**) and the model of lymphocyte CD4 + CD62L+ T cell transfer to C57BL/6 J mice (**c**). Colonic miR-146a expression was determined by RT-qPCR using U6 as reference gene. *p < 0.05 vs. control group (n = 5–8).

We assessed the expression of miR-146a in two different and widely used models of chemically induced colitis, namely TNBS colitis in rats and DSS colitis in mice. In addition, we evaluated miR-146a levels in Rag1^−/−^ mice transferred with CD4 + CD62L + T cells from regular donor mice i.e. lymphocyte transfer colitis. In the chemically induced colitis models the inflammatory reaction starts immediately or shortly after the administration of the colitogenic agent (TNBS or DSS) and is not strictly chronic (i.e. animals heal with time), while the transfer model is characterized by a slow, insidious onset (6–8 weeks) and a true chronic course. In addition, the latter model is lymphocyte driven, as is human inflammatory bowel disease (IBD), while both TNBS and DSS colitis develop virtually normally in the absence of lymphocytes^17,18^. The main characteristics of the three colitis models used in this study are shown in Table 1. Colonic expression of miR-146a as determined by RT-qPCR was upregulated in TNBS and DSS models but not in the transfer model (Fig. 6).

**Table 1:** Macroscopic parameters in several models of colitis.

	Rats		Mice			
	Control	TNBS	Control	Transfer	Control	DSS
Body weight gain (%)	9.2 ± 0.3	8.1 ± 3.2^+^	5.3 ± 0.6	1.8 ± 0.8^+^	5.8 ± 0.5	−8.1 ± 2.7^+^
Colonic weight/length ratio (mg/cm)	74.4 ± 9.7	221.5 ± 19.0^+^	21.6 ± 1.4	48.4 ± 5.5^+^	24.2 ± 0.2	43.3 ± 4.2^+^
Colonic damage extension (cm)	0 ± 0	3.5 ± 0.4^+^	—	—	—	—
Score	0 ± 0	13.0 ± 0.8^+^	—	—	—	—
Colonic damage score (AU)	—	—	0 ± 0	2.4 ± 0.4^+^	—	—

Data are mean ± SEM (n = 4–6).^+^p < 0.05 vs. control.

### Colonic expression of miR-146a is downregulated in TLR4 but not TLR2 knock out mice

To study the influence of bacterial antigen stimulation on the expression of miR-146a and immune activation through TLR2 and TLR4 we set up an experiment in which DSS colitis was induced in TLR2 and TLR4 knock out mice. Macroscopic characteristics of this colitis model are shown in Table 2.

**Table 2:** Macroscopic parameters in TLR2 and TLR4 knock out mice with DSS colitis.

			Mice			
	Control	DSS	KO TLR4	KO TLR4 DSS	KO TLR2	KO TLR2 DSS
Body weight gain (%)	0.9 ± 1.3	−4.9 ± 1.1^+^	3.3 ± 1.4	−7.1 ± 3.8^+^	7.6 ± 2.8	−1.7 ± 0.6^+^
Colonic weight/length ratio (mg/cm)	28.9 ± 2.3	34.4 ± 1.6^+^	22.1 ± 1.0	30.7 ± 2.1^+^	25.3 ± 3.6	30.9 ± 2.7

Data are mean ± SEM (n = 4). ^+^p < 0.05 vs. control.

**Figure 7:**
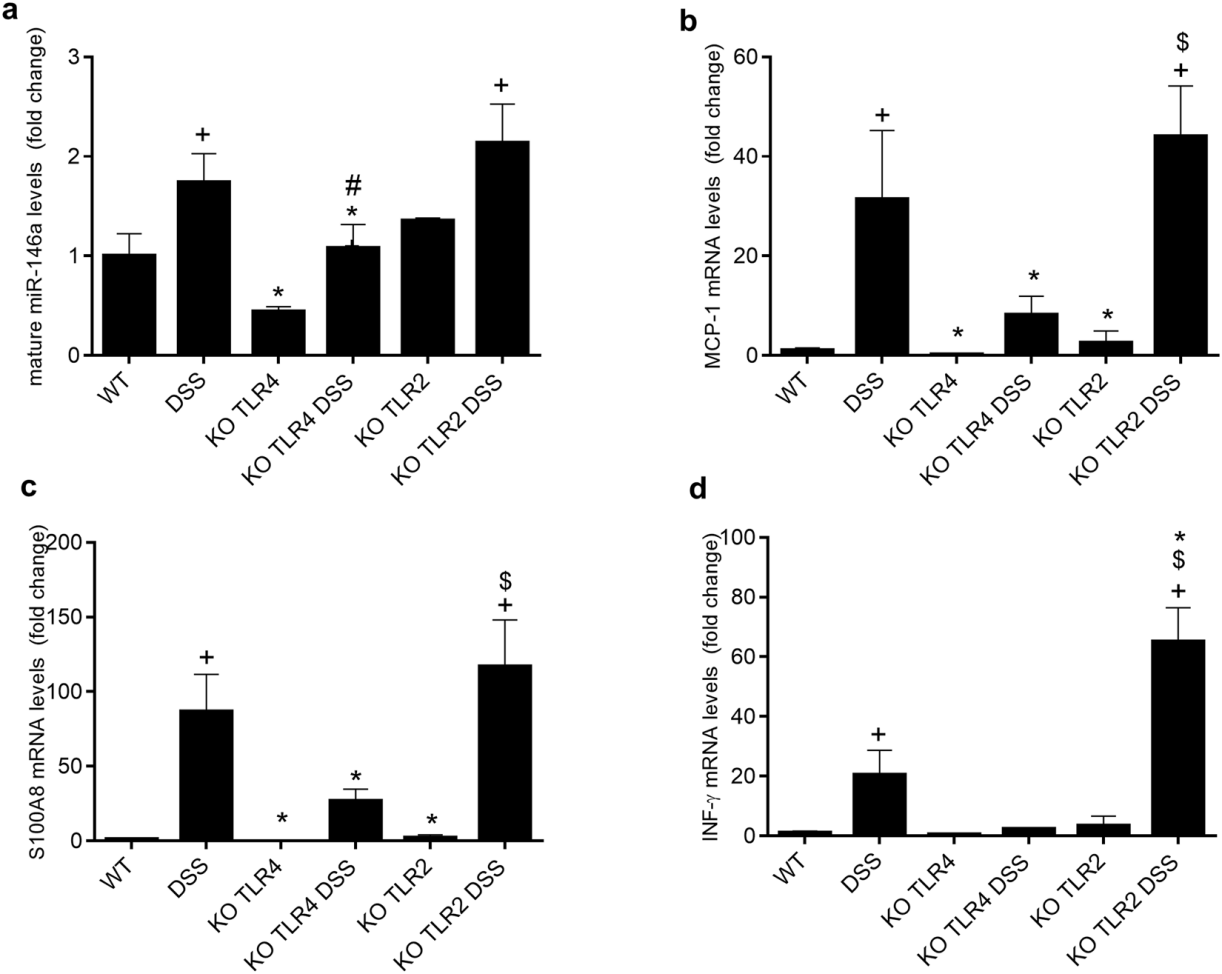
Colonic expression of mature miR-146a and inflammatory parameters in TLR2 and TLR4 kcnock out mice. Mice received DSS for seven days, and were then sacrificed. Colonic mature miR-146a (**a**), MCP1 (**b**), S100A8 (**c**) and INFγ (**d**) expression were determined by RT-qPCR using U6 as reference gene. ^+^p < 0.05 vs. Wild Type group (WT, n = 4); *p < 0.05 vs. DSS group (n = 4); ^#^p < 0.05 vs. TLR4 KO group (n = 4); ^$^p < 0.05 vs. TLR2 KO group (n = 4).

Consistent with the above results in cells, the absence of TLR4 was associated with downregulated miR-146a, as shown by the lower expression of this miRNA in the colon of TLR4 knock out mice when compared to wild type mice (Fig. 7). The inhibition of miR-146a expression was associated with reduced expression of MCP-1, S100A8 and IFNγ. As expected, DSS colitis significantly increased the expression of mature miR-146a, MCP-1, S100A8 and IFNγ. However, the induction of these genes was attenuated in TLR4 knock out mice, indicating a lower response to colitis induction. Interestingly, these effects on TLR4 knock out mice were specific since the absence of TLR2 had no effect on miR-146a expression and actually resulted in an increase in the expression of MCP-1, S100A8 and IFNγ.

### miR-146a is overexpressed in differentiated intestinal epithelial cells

**Figure 8:**
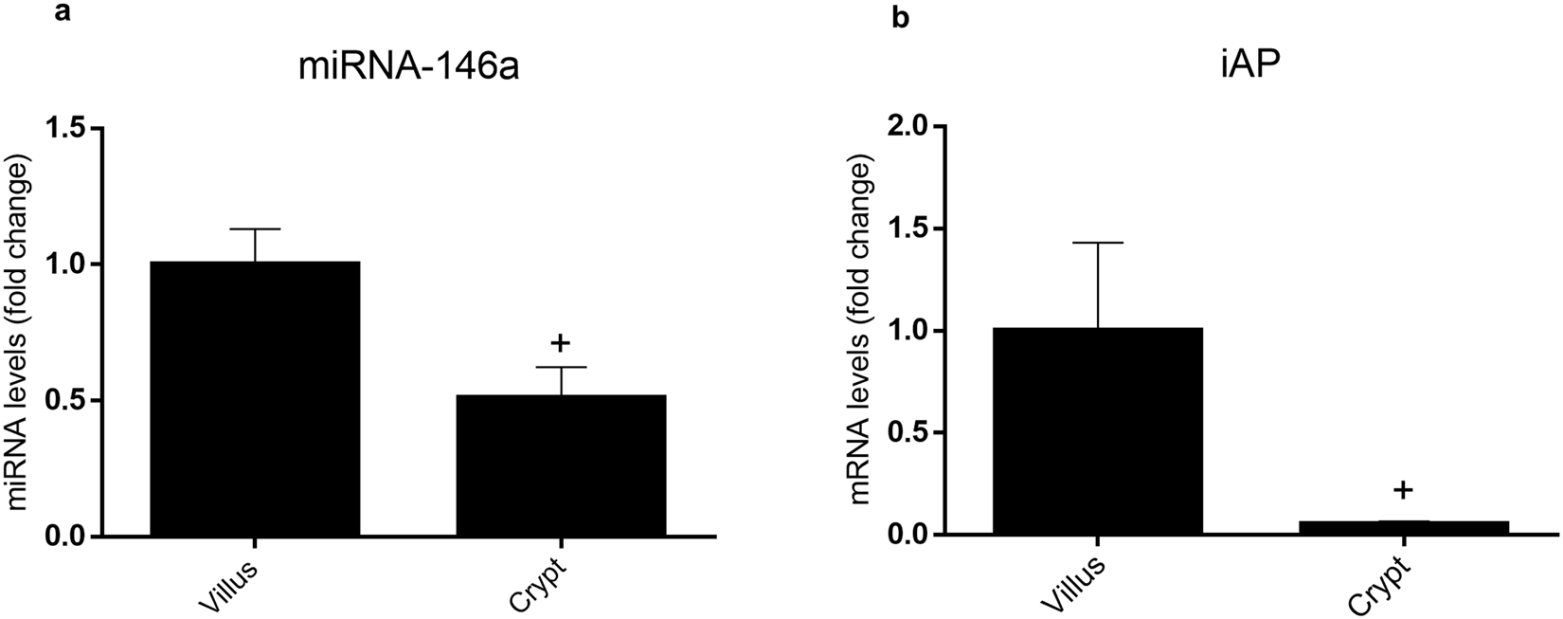
miRNA-146a and iAP expression in crypts and villus IECs of mice jejunum. Expression of miRNA-146a (**a**) and iAP (**b**) in crypts and villus IECs of mice jejunum were studied by RT-qPCR, using U6 and 18 S for miRNA-146a and iAP as reference genes, respectively. ^+^p < 0.05 vs. IECs (n = 4).

Crypts and villus IECs from mouse jejunum were isolated and the expression of miR-146a was measured by RT-qPCR (Fig. 8a). Levels were found to be significantly upregulated in villus when compared to crypt cells. As expected, the expression of iAP, a marker of IEC differentiation, followed the same pattern (Fig. 8b). In order to assess the purity of villi and crypt preparations, we determined the expression of villin (a brush-border specific protein,), LGR5 (Leucine-rich repeat-containing G-protein coupled receptor 5, biomarker of intestinal stem cells) and CD3 (cluster of differentiation 3, part of the T cell receptor) by qRT-PCR (see Supplementary Fig. 1). Our results indicate that the expression of villin was significantly increased (11.5 ± 4.4 fold, p < 0.05 (n = 5)) in villus cells when compared to crypt cells. Ct for CD3 and LGR5 amplification in crypts was 34.0 ± 1.1 and 37.6. ± 1.2, respectively, and no amplification was detected for these markers in villus cells.

## Discussion

Interactions between the intestinal microbiota and the innate immune system contribute to regulate intestinal homeostasis. miRNAs control gene expression and among them miR-146a is known to be produced in response to bacterial LPS and cytokines to regulate the immune response. In fact, miR-146a is expressed in hematopoietic cells and is known to exert protective anti-inflammatory effects in systemic compartments such as the bone marrow, the spleen and lymph nodes^17–20^. Nevertheless, its role in the inflamed intestine remains controversial and its contribution to the regulation of the innate immune system by IECs has been poorly assessed. Although miR-146a has been reported to play an anti-inflammatory role, even at the intestinal level (it protects against intestinal inflammation induced by ischemia/reperfusion injury^16^), recent studies have shown that it also increases susceptibility to DSS colitis in mice^21^. The explanation given to these differences has been based on the ability of miR-146a to repress pro-inflammatory genes (protecting against inflammation) but also those that fortify the intestinal barrier, thereby potentially increasing susceptibility to colitis as the result of enhanced contact of immune cells with intestinal bacterial antigens^21^. In order to further explore the effect of miR-146a in intestinal inflammation we have used three different animal models of colitis with differential effects on the intestinal barrier. The TNBS and the DSS models chemically induce colitis and, although the exact mechanism by which they act is still incompletely understood, in both cases a severe disruption of gut mucosal barrier integrity is required, allowing luminal antigens access to the lamina propria and the proinflammatory cells within^22^. The two models differ in other respects. In the former, TNBS acts as a hapten and elicits a delayed hypersensitivity response in the mucosa after reaction with resident proteins. This is an immune mechanism and accordingly it is amenable to modulation by immunological maneuvers such as induction of tolerance by feeding TNBS haptenated proteins^23^ or colitis reactivation by parenteral readministration of TNBS^24^. However, in order to get access to the mucosa, TNBS is administered in an alcoholic solution, which produces an epithelial lesion. Although ethanol elicits an inflammatory reaction per se, this is short lived in the absence of TNBS. Thus the protracted, semi-chronic colitis in this model is accounted for by TNBS rather than ethanol. In DSS colitis the main mechanism is epithelial toxicity, which is reproducible *in vitro* and develops slowly over several days. The result is enhanced access of the mucosal immune system to luminal antigens and microorganisms, which ultimately gives rise to colitis^25^.

The third model used is based on the transfer of naïve T lymphocytes to immune deficient mice, which are repopulated progressively by the transferred lymphocytes and develop mild colitis after several weeks^26,27^. In the three models, and actually in virtually all the experimental IBD models available, the presence of the intestinal microbiota is needed for inflammation to develop. However, the compromise of the intestinal barrier is much lower in the transfer model than in TNBS and DSS colitis^28^. Our data indicate that miR-146a is increased significantly in the DSS and the TNBS models, but not in lymphocyte transfer colitis. Hence there is a correlation between barrier disruption and activation of the innate immune system and the induction of miR-146a. Moreover, our results indicate that specific stimulation of TLR4 receptors is involved in the induction of miR-146a expression. In fact, the induction of colitis by DSS in the absence of TLR4, but not in the absence of TLR2, downregulated miR-146a expression. Residual expression of miR-146a indicates that other pathways are at play, as expected.

The importance of bacterial signals for miR-146a induction is supported by *in vitro* data obtained in IECs. TLRs are highly involved in the crosstalk between the innate immune system and the microbiota^4^, and IECs express TLRs and produce cytokines in response to their activation. In addition, IECs respond to intestinal inflammation producing cytokines. Therefore these cells are increasingly being considered innate immune cells. The activation of TLRs by their ligands induce the expression of miR-146a in a variety of peripheral tissues^16,19^ and in different cell types (primary macrophages, neutrophils and dendritic cells)^29^. IL-1β and TNF are also able to induce miR-146a in THP-1 cells^29,30^. Here, we demonstrate that these effects are also produced in IECs. Namely, TLR4 and TLR5 ligands as well as TNF induced the expression of miR-146a in IEC18 cells^30^. IL-1β had the same effect in Caco-2 cells. On the other hand, no effect was observed for TLR9, TLR2 or NOD2 ligands, possibly because of low receptor expression, as they did not elicit a positive cytokine response. TLR4 involvement is supported by the downregulation of miR-146a in TLR4 KO mice with DSS colitis compared with WT mice. This was a specific finding since TLR2 KO mice displayed no defect in miR-146a expression. In agreement with our findings, a study in neonatal intestine indicated that TLR4 expression is induced in the intestine during the neonatal period, with the subsequent upregulation of miR-146a^28^. Thus we further studied the involvement of TLR4 and MyD88 (an adaptor protein involved in the signal transduction of all TLRs except TLR3). As expected, the upregulation of miR-146a after LPS addition was abolished when TLR4 or MyD88 were silenced in IEC18 cells, further sustaining the hypothesis that miR-146a expression is positively linked to the TLR4-MyD88 pathway. Moreover, we studied downstream signaling and confirmed the involvement of NFκB and AKT, but not MAP kinases.

In terms of biological significance, miR-146a has been proposed as a mediator of innate immune tolerance in the intestine, contributing to prevention of inflammation induced epithelial damage. Overexpression of miR-146a confers tolerance against stimulation with LPS in mouse intestine^28^ as well as against IL-1β, as recently shown in THP-1 monocytes^30^. In our study, we overexpressed miR-146a in IEC18 cells and found a decreased production of both MCP-1 and GROα when the cells were stimulated with LPS. The same effect was observed on MCP-1 and IL-8 when Caco-2 cells were stimulated with IL-1β. Therefore, miR-146a overexpressed in IEC cells contributes to innate immune tolerance by inhibiting the expression of proinflammatory cytokines. Hence IEC stimulation with LPS/IL-1β appears to activate both positive and negative signals toward cytokine secretion. In a similar fashion, it has been shown that THP-1 cells stimulated with LPS exhibit reduced TNF levels in the culture medium measured a few hours after stimulation, coinciding with miR-146a expression^15^. Our data also indicate that miR-146a expression is associated with IEC differentiation, suggesting heightened immune tolerance in surface enterocytes. Thus when crypt cells (undifferentiated as assessed by the low expression of alkaline phosphatase and villin and the expression of LGR5) and villus cells (differentiated) of the small intestine of mice were compared, miR-146a was found to be overexpressed in the latter. Furthermore, results of our laboratory not included in this article indicate that the differentiation of Caco-2 cells also induces the expression of miR-146a (fold change = 5.2). However, miR-146a upregulation does not seem to influence cell differentiation or proliferation (data not shown). Our data are reminiscent of miR-146a upregulation in immune cells upon maturation^19^.

In summary, we have expanded the current view of miR-146a in the intestine by establishing that it is induced in IEC in response to both differentiation and to a variety of proinflammatory stimuli, including LPS acting via the TLR4-MyD88-AKT/NFκB pathway. The latter appears to be especially relevant in experimental colitis. miR-146a dampens IEC immune activation, suggesting that it may contribute to immune tolerance in the gut. These findings underscore the importance of miR-146a regulation in the crosstalk among IECs, the intestinal microbiota and inflammatory stimuli.

## Materials and Methods

### Materials and reagents

Except where indicated, all reagents and primers were obtained from Sigma-Aldrich (Barcelona, Spain).

### Animals

Female Wistar rats (175–225 g), obtained from Harlan (Barcelona, Spain), were housed in makrolon cages and maintained in air conditioned animal quarters with a 12 h light–dark cycle. They were provided with free access to autoclaved tap water and a standard chow diet (Panlab A04, Panlab, Barcelona, Spain).

Female C57BL/6 J wild type, B6.B10ScN-Tlr4lps-del/JthJ (Tlr4 KO), B6.129-Tlr2 < tm1Kir >/J (Tlr2 KO) and Rag1^−/−^ mice were obtained from Jackson Laboratory (CA, USA). Animals were maintained at the unit of animal research (Biomedical Research Center, University of Granada, Granada, Spain) in air-conditioned animal quarters with a 12 h light-dark cycle. Animals were housed per groups in specific Pathogen-Free (SPF) conditions, in Individual Ventilated Cages (IVC) with an air insufflation and exhalation system with dual filter (pre-filter and HEPA filter), and were given free access to autoclaved tap water and food (Harlan-Teklad 2014, Harlan Ibérica, Barcelona, Spain).

This study was carried out in accordance with the Directive for the Protection of Vertebrate Animals used for Experimental and other Scientific Purposes of the European Union (86/609/EEC) and was approved by the Ethical Committee of the University of Granada (reference n°150-2007).

### Models of experimental colitis

Three different models of colitis were used:

- Trinitrobenzenesulfonic acid (TNBS) model of rat colitis: colitis in rats was induced according to the method described by Morris *et al*.^31^ with minor modifications. Briefly, rats were fasted overnight and anaesthetized with isoflurane. Under these conditions, rats were given 10 mg of TNBS dissolved in 0.25 ml of 50% ethanol (v/v) by means of a Teflon cannula inserted 8 cm through the anus. Rats from the non-colitic (control) group received 0.25 ml of PBS. Rats were kept in a head-down position for an additional 30 s until they were recovered from anesthesia, and returned to their cage. Occurrence of diarrhea (perianal fur soiling) was recorded. Animals were killed by cervical dislocation, after 5 days.
- DSS model of mice colitis: DSS colitis was induced by addition of DSS 2.5% (w/v) (MP Biomedicals, Santa Ana, California, USA), to drinking water for 7 days. The status of mice was monitored by general examination and specifically by means of the disease activity index (DAI), a combined score for weight loss, diarrhea, and hematochezia, which are 3 main signs of pathology in this model^32^.
- Lymphocyte transfer colitis: chronic colitis was induced by the transfer of a naïve lymphocyte population from female wild type C57BL/6 J donor mice to immunodeficient (Rag1^−/−^) female mice on the same genetic background^27^. Naïve T cells were obtained from the spleen of donor mice by mechanical dissection and magnetic enrichment using a protocol with magnetic microbeads (CD4 + CD62L + T cell isolation kit for mouse, Miltenyi Biotec^®^, Bergisch Gladbach, Germany). Once purified, cells were resuspended in PBS, which was used as vehicle for intraperitoneal injection into immunodeficient recipient mice. Control animals were injected with PBS alone, while colitic animals were injected with 100 µl of PBS containing 10^6^ CD4 + CD62L + cells per mouse. Animals were sacrificed by cervical dislocation 8 weeks after cell transfer, when there were signs of significant disease according to a disease activity index based on body weight loss, presence of blood in faeces, rectal prolapse and diarrhea.

### Cell lines

Caco-2 cells (ECACC 09042001, human colon adenocarcinoma cells, passages 30–50), plus IEC18 cells (ECACC 88011801 nontransformed rat small intestinal epithelial, passages 25–50) were obtained from the ECACC. They were cultured in DMEM supplemented with heat-inactivated FBS (10% v/v), 100 IU/ml penicillin, 0.1 mg/ml streptomycin, 2.5 µg/ml amphotericin and 2 mM L-glutamine, in a humidified 5% CO_2_ atmosphere at 37 °C. Both cell lines were used since they express different receptors and, as a consequence, they respond differently to stimuli. Because Caco-2 cells used did not express TLR4, experiments in which the response to LPS was studied were carried out with IEC18 cells^33^.

### *In vitro* assays with cell lines

All experiments were carried out with confluent monolayers except experiments involving transfection of small RNA mimic of miR-146a, which were 60–80% confluent at transfection. The reagents used were: LPS (*Escherichia coli* 055:B5, 1 µg/ml), TNF (10 ng/ml), IL-1β (10 ng/ml), flagellin (100 ng/ml) (InvivoGen, San Diego, California, USA), cytosine phosphate guanine (CpG) DNA (50 nM) (HyCult Biotech, The Netherlands), peptidoglycan (PDG, 2 µg/ml) and muramyldipeptide (MDP, 5 µg/ml). For the cytokine secretion assays, Caco-2 and IEC18 cells were cultured until confluence and stimulated with the different proinflammatory cytokines or bacterial components for 24 h. Vehicle treated cells were used as negative control. Afterwards, the supernatants were collected, cleared by centrifugation at 4000xg for 5 min, and kept at −80 °C until measurement. IL-8, MCP-1 and GROα were determined by ELISA kits. GROα ELISA kit was obtained from R&D System (Abingdon, UK) and MCP-1 and IL-8 ELISA kits were obtained from Beckton Dickinson Biosciences (Madrid, Spain).

In order to explore downstream signaling pathways, IEC18 confluent monolayers were exposed to Bay 11-7082 (10 μM), a selective inhibitor of IκB-α phosphorylation that blocks the NF-κB signaling pathway, or wortmannin (1 μM), an inhibitor of phosphatidylinositol 3 kinase (PI3K) phosphorylation that inhibits the Akt signaling pathway. The p38 MAPK inhibitor SB203580 (10 μM), the ERK1/2 MAPK inhibitor PD98059 (10 μM) (EMD Millipore, Billerica, Massachusetts, USA), or the JNK inhibitor SP600125 (10 μM) were also added to the cell cultures to selectively inhibit the phosphorylation of these MAPKs. Cytokine secretion was measured as above.

### TLR4 and MyD88 gene knockdown in IEC18 cells

IEC18 cells were pretreated with shRNA specific for MyD88 or TLR4 (Santa Cruz Biotechnologies, Heidelberg) for gene knockdown, following the manufacturer´s instructions. Briefly, IEC18 cells were plated on six well plates and grown for 24 h until 50% confluence. Before infection, IEC18 medium was supplemented with polybrene 5 µg/mL and cells were incubated for 10 h. Control, MyD88 and TLR4 shRNA lentiviral particles were also separately pretreated with polybrene 5 µg/mL (Santa Cruz Biotechnogy, Heidelberg) for 30 min, added to the culture medium and incubated overnight. On the third day, lentiviral particles containing medium was substituted and the cells were cultured until confluence for 24 h. Finally, IEC18 cells were split (1:5), cultured again for 24 h, and selected with a range of 5–10 µg/mL of puromycin dihydrochloride.

### Transfection of IEC18 and Caco-2 cells with small RNA mimic of miR-146a

IEC18 and Caco-2 cells were used at 60–80% confluency. Cells were cultured in 24-well plates with DMEM supplemented medium with heat-inactivated FBS (10% v/v), 100 IU/ml penicillin, 0.1 mg/ml streptomycin, 2.5 µg/ml amphotericin and 2 mM L-glutamine, in a humidified 5% CO_2_ atmosphere at 37 °C. Lipofectamine RNAiMAX (ThermoFisher, Waltham, MA, USA) was used to transfect cells lines IEC18 and Caco-2 with hsa-miR-146a-5p mirVana mimic or nontargeting negative control (miR-NC) oligonucleotides, at 30 and 60 nM respectively (Life Technologies, Carlsbad, CA) according to the manufacturer’s instructions. The mirVana mimics (Ambion/ThermoFisher) are double-stranded oligonucleotides mimicking mature microRNA.

### Crypt and IECs isolation from mouse jejunum

The method for crypt isolation was taken from protocols described previously^34^. In short, the jejunum from C57BL/6 J mice was opened longitudinally, and washed with cold PBS. To isolate crypts, the villi were scraped with a coverslip and the tissue was chopped into pieces of approximately 5 mm, and further washed with cold PBS. These fragments were incubated in 2 mM EDTA with PBS for 30 min at 4 °C and subsequently washed and dispersed in PBS by repetitive pipetting. Crypts were then obtained by centrifugation and saved for later RNA extraction. To isolate villi IECs we followed the method described by Weigmann *et al*.^34^ with some modifications. In short, the jejunum was washed first with PBS, opened longitudinally and washed again with a solution of HBSS (Hanks balanced salt solution) with 1 mM DTT. The tissue was cut into fragments of approximately 5 mm and the fragments were subjected to two successive incubations in HBSS with 5% FBS and 2 mM EDTA. IECs were further purified by separation in easycoll (Biochrom, Cambridge UK) 80:40 (v/v).

### RNA isolation & analysis of gene expression by reverse transcriptase-quantitative PCR

Total RNA from rat or mouse tissues and Caco-2 or IEC18 monolayers was isolated with Trizol^®^ reagent using the method described by the manufacturer (ThermoFisher, Waltham, MA, USA) and the quantity and integrity of RNA were assessed by spectrophotometry (absorbance ratio 260/280 nm) and 1% (w/v) agarose gel electrophoresis, respectively. One microgram was retrotranscribed (iScript, BioRad, Alcobendas, Spain) and specific RNA sequences were amplified with CFX ConnectTM Real-Time PCR Detection System (Biorad Laboratories®, California, EEUU) using the primers shown in Table 3. For normalization we used U6 snRNA (a small nuclear RNA (snRNA)) as an endogenous control for pre-mir-146a analysis. 18 S was used for the rest of the genes.

**Table 3:** DNA sequence of primer pairs used in PCR assays in human, rat and mice tissue.

Gene name	Primer sequence forward (5′−3′)*	Primer sequence reverse (5′−3′)*	Annealing temperature (°C)	Efficiency(%)
*Rattus norvegicus* pre-mir-146A	GCTCTGAGAACTGAATTCCATGGGT	ATGACGATAGAGCTATCCCAGC	61	72.1
*Mus musculus* pre-mir146A	GCTCTGAGAACTGAATTCCATGGGT	AGCTGAAGAACTGAATTTCACAGGTC	61	81.7
*Homo sapiens*, *Rattus norvegicus* and *Mus musculus* RNU6-1	GCTCGCTTCGGCAGCACATATACTAA	ACGAATTTGCGTGTGTACTCCTTGCG	69	91.1
*Homo sapiens* mature MIR146a	UGAGAACUGAAUUCCAUGGGUU	Universal primer (Quiagen Cat. N° 1046471)	55	90.6
*Mus misculus* mature Mir146a	UGAGAACUGAAUUCCAUGGGUU	Universal primer (Quiagen Cat. N° 1046471)	55	113
*Rattus norvegicus* mature Mir146a	UGAGAACUGAAUUCCAUGGGUU	Universal primer (Quiagen Cat. N° 1046471	55	90.3
*Control snRNA* RNU6B	Quiagen Cat. N° MS00033740	Quiagen kit Cat. N° MS00033740	55	103.5
*Mus musculus* Ifng	GCTCTGAGACAATGAACGCTACAC	TTCTTCCACATCATGCCACTTGAG	59.2	100.7
*Mus musculus* Ccl2	CAAGATGATCCCAATGAGTAG	TTGGTGACAAAAACTACAGC	60	96.5
*Mus musculus* S100a8	GCCCTCTACAAGAATGACTTCAAG	ATCACCATCGCAAGGAACTCC	60	91.4
*Mus musculus* Alpi	CATGGACATTGATGTGATCC	AGACTGGTTACTGTCACTTG	60	96.7
*Mus musculus* 18 s	ACACGGACAGGATTGACAGATTG	GCCAGAGTCTCGTTCGTTATCG	60	88
*Mus musculus* Hprt	AGGGATTTGAATCACGTTTG	TTTACTGGCAACATCAACAG	60	97.3
*Mus musculus* Ppib	TGGAGATGAATCTGTAGGAC	CAAATCCTTTCTCTCCTGTAG	60	100.4
*Mus musculus* Vil1	TATGGAGGATCGAGGCTATG	AGGTAGTCATCCATCTGTGT	60	98.9
*Mus musculus* Lgr5	AGAACACTGACTTTGAATGG	CACTTGGAGATTAGGTAACTG	58	116.2
*Mus musculus* Cd3e	ATCTTGGTAGAGAGAGCATTC	CCCATTTTAAGTTCTCGTCAC	60	109.4
*Rattus norvegicus* Tlr2	AAAAACTGCTGAGATTTTGC	TACTAACATCCAACACCTCC	60	84.6
*Rattus norvegicus* Tlr5	AAAAGCAGCAGTATTTGAGG	ACTAGGAAACCGTCCTTATG	60	86.3
*Rattus norvegicus* Tlr9	CCTTTGGTTTCCAGTAACATC	CAGAAGTCCATATTCACAGTTC	60	106
*Rattus norvegicus* Nod2	AACCTCAGCCATAACATCC	ACTTCCAGCAGTAAGTCTAC	60	94
*Rattus norvegicus* Tlr44	ACCTAGATCTGAGCTTCAAC	TTGTCTCAATTTCACACCTG	60	113.8
*Rattus norvegicus* 18 s	GGACACGGACAGGATTGAC	TCGCTCCACCAACTAAGAAC	60	96.3

For detection and quantification of mature miR-146a we used the miScript PCR System (Quiagen, Hilden, Germany). With this method, cDNA is prepared using the miScript II RT Kit and subsequently, real-time PCR is performed using a miScript Primer Assay (forward primer) specific for rat or mice mature miR-146a and the miScript SYBR Green PCR Kit, which contains the miScript Universal Primer (reverse primer). For normalization of real-time PCR results using the miScript PCR System we used the miScript PCR Control snRNA RNU6B (RNU6-2) that enables normalization of real-time PCR results in miRNA quantification studies using this system.

### Statistical analysis

All results are expressed as mean ± standard error of the mean (SEM). Results of *in vitro* experiments are representative of at least two independent experiments. Differences among means were tested for statistical significance using Student’s t-test when two groups were compared or by one-way ANOVA and a posteriori Fisher-LSD test. Analyses were carried out with the SigmaStat 3.5 program (Jandel, San Jose, CA, USA). Differences were considered significant at *p* < 0.05.

## Electronic supplementary material

Supplementary Figure 1
