# Supplementary material for: miR-146a regulates the crosstalk between intestinal epithelial cells, microbial components and inflammatory stimuli
Source: Sci Rep. 2018 Nov 26;8:17350. doi: 10.1038/s41598-018-35338-y (PMC6255912; doi:10.1038/s41598-018-35338-y)
Supplement: Supplementary file 1 — Supplementary Figure 1 [file 41598_2018_35338_MOESM1_ESM.pdf]

miR-146a regulates the crosstalk between intestinal epithelial cells, microbial components and inflammatory stimuli

Andrea Anzola, Raquel González, Reyes Gámez-Belmonte, Borja Ocón, Carlos J. Aranda, Patricia Martínez-Moya, Rocío López-Posadas, Cristina Hernández-Chirlaque, Fermín Sánchez de Medina, Olga Martínez-Augustin

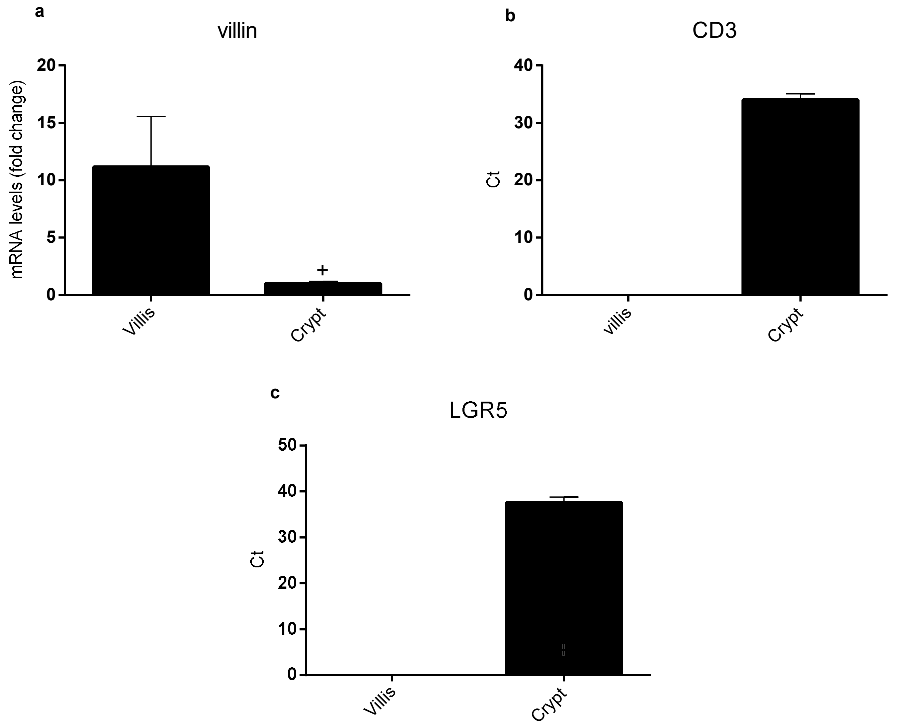

Supplementary Figure 1. Villin, LGR5 and CD3 expression in crypts and villi IECs of mouse jejunum. Expression of villin (a), LGR5 (b) and CD3 (c) in crypt and villus IECs of mouse jejunum were measured by RT-qPCR. 18S was used as reference gene. $^{+}p< 0.05$ (n=5).
